# Promiscuous Enzyme Activity as a Driver of Allo and Iso Convergent Evolution, Lessons from the β-Lactamases

**DOI:** 10.3390/ijms21176260

**Published:** 2020-08-29

**Authors:** Vivek Keshri, Eric Chabrière, Lucile Pinault, Philippe Colson, Seydina M Diene, Jean-Marc Rolain, Didier Raoult, Pierre Pontarotti

**Affiliations:** 1 Aix-Marseille Univ IRD, APHM, MEPHI, IHU Méditerranée Infection, 19-21 Boulevard Jean Moulin, 13005 Marseille, France; vivek.bioinfo@gmail.com (V.K.); eric.chabriere@univ-amu.fr (E.C.); lucile.pinault@gmail.com (L.P.); philippe.colson@univ-amu.fr (P.C.); seydina.diene@univ-amu.fr (S.M.D.); jean-marc.rolain@univ-amu.fr (J.-M.R.); didier.raoult@gmail.com (D.R.); 2 SNC5039 CNRS, 19-21 Boulevard Jean Moulin, 13005 Marseille, France

**Keywords:** β-lactamase, convergent evolution, antibiotic resistance

## Abstract

The probability of the evolution of a character depends on two factors: the probability of moving from one character state to another character state and the probability of the new character state fixation. The more the evolution of a character is probable, the more the convergent evolution will be witnessed, and consequently, convergent evolution could mean that the convergent character evolution results as a combination of these two factors. We investigated this phenomenon by studying the convergent evolution of biochemical functions. For the investigation we used the case of β-lactamases. β-lactamases hydrolyze β-lactams, which are antimicrobials able to block the DD-peptidases involved in bacterial cell wall synthesis. β-lactamase activity is present in two different superfamilies: the metallo-β-lactamase and the serine β-lactamase. The mechanism used to hydrolyze the β-lactam is different for the two superfamilies. We named this kind of evolution an allo-convergent evolution. We further showed that the β-lactamase activity evolved several times within each superfamily, a convergent evolution type that we named iso-convergent evolution. Both types of convergent evolution can be explained by the two evolutionary mechanisms discussed above. The probability of moving from one state to another is explained by the promiscuous β-lactamase activity present in the ancestral sequences of each superfamily, while the probability of fixation is explained in part by positive selection, as the organisms having β-lactamase activity allows them to resist organisms that secrete β-lactams. Indeed, an organism that has a mutation that increases the β-lactamase activity will be selected, as the organisms having this activity will have an advantage over the others.

## 1. Introduction: The Concepts: Iso and Allo-Convergent Evolution, Evolutionary Shift, and Maintenance of Apomorphies

In order to gain a finer-scale understanding of the dynamics of convergence, we proposed to use the concepts and corresponding terms “iso-convergent” and “allo-convergent” evolution [1]. Iso-convergent traits have converged from the same ancestral state (traditionally “parallel evolution” but parallel evolution also has a different meaning), whereas allo-convergent traits have converged from different ancestral states. In the case of iso-convergent evolution, because we could define the ancestral and the derived state, it could be possible to modelize the process that allows the shift. The probability of going from one state to another is better explained in the case of amino acid substitutions, where the evolutionary model has been well studied. The substitution (shift from an ancestral to a derived state) depends on two factors: the mutation rate and the fixation rate. Regarding the mutation rate, some shifts are more probable than others. For example, the transition is more probable than transversion, and in the genomes of some vertebrates’ taxa, another important form of mutation bias involves changes at CpG dinucleotides. If the DNA nucleotide cytosine (C) is immediately 5′ to a guanine (G) on the same coding strand, then depending on methylation status, point mutations at both sites occur at an elevated rate relative to mutations at the non-CpG site [2]. Regarding the fixation rate, besides the importance of positive selection, an important factor has to be taken into account: the pleiotropy. Indeed, amino acid substitution usually has pleiotropic effects on protein biochemistry. A substitution that improves one aspect of a protein function may also compromise other structural or functional properties. Within a set of mutations that functionally have the same effects on the phenotype, the mutations that display the lower deleterious pleiotropic effect should have a higher fixation probability [3]. The dichotomy of mutation rate and fixation rate can be found at higher biological levels. However, usually at higher biological levels, scholars discussed constraint and selection [4], whereas the probability of shift (mutation rate) has not been discussed. For example, Losos (2011) [4] pointed out that the wings allowing flight in vertebrates have been built convergently in different ways in birds, pterosaurs, and bats. In all these cases, the wings represent modified forelimbs. The combination of wings and forelimbs, in theory, could be very useful but is not found in real life. The author concluded that this was due to a lack of constraints. We underline here that, besides the constraints, the probability of modifying forelimbs to obtain wings is likely higher than that of starting from nothing. Note that the same reasoning could be applied to the DDE transposon co-option as sequence-specific recombination activating systems that evolved via iso-convergent evolution. This is explained many times in part by the fact that the biochemical shift from a transposase to a sequence-specific recombination activating endonuclease is an easy evolutionary step [5]. We think that it is mandatory to clarify the participation of the shift and fixation probability and we will show that this clarification is important regarding the process of iso-convergent and allo-convergent evolution. Furthermore, we describe a property that increases the probability of this shift: the enzymatic promiscuity.

## 2. Enzyme Convergent Evolution via Allo-Convergent and Iso-Convergent Evolution: The Case of β-Lactamases

### 2.1. Allo-Convergent Evolution of Enzyme Function: The Case of β-Lactamases

The allo-convergent evolution of enzyme function results in two distinct mechanisms. First, non-homologous enzymes deliver the same transformation as expressed by the same four-digit enzyme commission (EC) number [6,7], but with a different mechanism. Second, the other case of allo-convergent evolution corresponds to the one where enzyme transformation is realized by a similar disposition of residues in the active site; the active site then occurs in an independent manner [6,7]. The enzyme commission (EC) number is a numerical classification scheme for enzymes based on the catalyzed chemical reactions. In enzymes that exhibit multispecificity or substrate ambiguity, the EC number for the various substrates should be the same/differ only by the fourth digit between enzymes of the same class. Catalytic promiscuity refers to cases in which the EC numbers of the various substrates and reactions catalyzed by the same enzyme differ in the second or the third digits that refer to different chemistries and different classes of substrates, or even by the first digit that indicates a completely different reaction category [8].

The β-lactamases that correspond to the former case have the same EC number (3.5.2.6) and they correspond to two distinct families: the serine β-lactamases (class A/C/D) and the metallo-β-lactamases (class B) superfamilies, therefore with two distinct ancestors.These two superfamilies can hydrolyze the antibacterial β-lactams. The β-lactams inhibit penicillin-binding proteins, which are involved in the cell wall synthesis of bacteria, by performing cross-linking of peptide chains to form peptidoglycan. The inhibition is performed by acylating an active-site serine that is essential for penicillin-binding protein activity [9].

The mechanisms by which the two superfamilies of β-lactamase perform the hydrolysis, and thus the resistance to β-lactam, are different. In the case of the A, C, and D serine β-lactamase family, the hydrolysis occurs through the formation of an acyl-enzyme with an active-site serine and in the case of metallo-β-lactamases this occurs via a hydrolytic reaction facilitated by one or two essential zinc ions in the active sites. The independent evolution of the same function, most often using different mechanisms, is well documented for proteins of different families of nonhomologous enzymes [7].

The case of iso-convergence has been less documented, one of the best-described examples being the “HAD (haloacid dehalogenase) superfamily” of proteins [10]. In the case of the different families of β-lactamase [11], the iso-convergent evolution of β-lactamases was found.

While Keshri et al. [11] focused on the convergent (iso-convergent) evolution of the β-lactam’s specificities in the four different classes of β-lactam, we will focus our analysis on the independent evolution of the generic β-lactamase function in regard to the evolution of the metallo-β-lactamases superfamily and the serine-β-lactamases superfamily.

### 2.2. Iso-Convergent Evolution of the Metallo-β-Lactamases Functional Family

#### Evolutionary Analyses of the Metallo-β-Lactamases Family

In general, a superfamily based upon a structural fold is organized in structural families. Each family could have its own function. However, this is not always the case; this is due, as we will see, to iso convergent evolution. This is why we use the terms functional family and structural family since these two sentences have a different meaning.

The metallo-β-lactamase family is a superfamily, the superfamily being defined based on a structural fold and the eponymic family being the metallo-β-lactamases family. At least 23 other functional families have been identified including enzymes involved in DNA and RNA nucleotide processing, detoxification, quorum quenching, and pesticide hydrolysis. Most of these functions involve hydrophilic reactions and target different substrates with different chemical properties, for example phosphodiester, phosphotriester, choline phosphoester, thiol ester, sulfonate ester, and β-lactam bond. Other functions involve non-hydrolytic reactions such as nitric oxydoreduction and sulfur dioxygenation as well as non-enzymatic functions [12,13].

**Figure 1:**
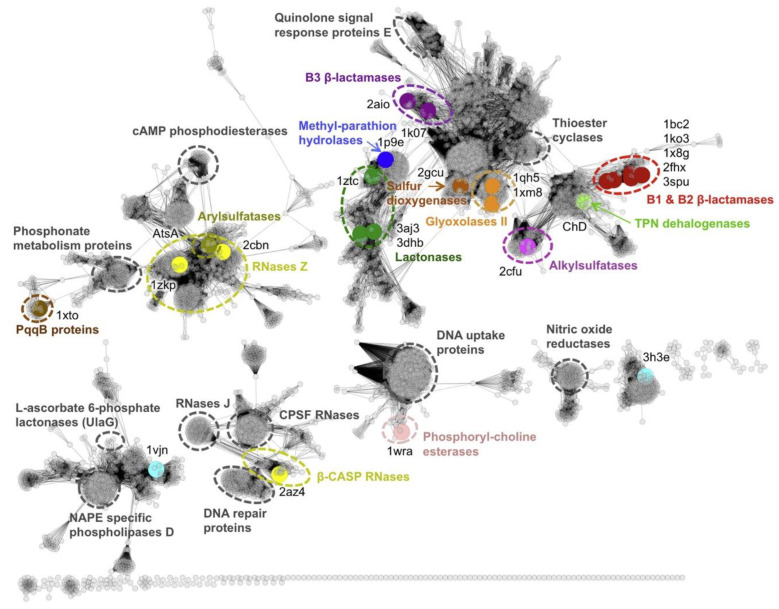
Sequence similarity network of metallo-β-lactamase (MBL) superfamily members. A total of 6233 sequences (nodes) and lines (edges) show a sequence relationship at a BLAST e-value cutoff of 1 × e^−14^. Large colored nodes show sequences that were experimentally characterized with colored broken circles indicating their approximate functional family cluster. Pale-blue-colored nodes (PDB IDs 1vjn and 3h3e) are experimentally characterized sequences with unknown function and from clusters with unknown function. The functional family clusters of three experimentally characterized sequences have not been encircled due to the small number of functional homologs (methyl-parathion hydrolase and TPN dehalogenase) and annotation ambiguity (sulfur dioxygenase). Gray broken circles indicate functional sequence clusters that have been experimentally characterized and reported in the literature but have not been included in this study. For unassigned gray sequence clusters (not encircled), no confident functional information could be retrieved from the databases or literature. This figure has been reprinted with permission from Baier and Tokuriki [13].

**Figure 2:**
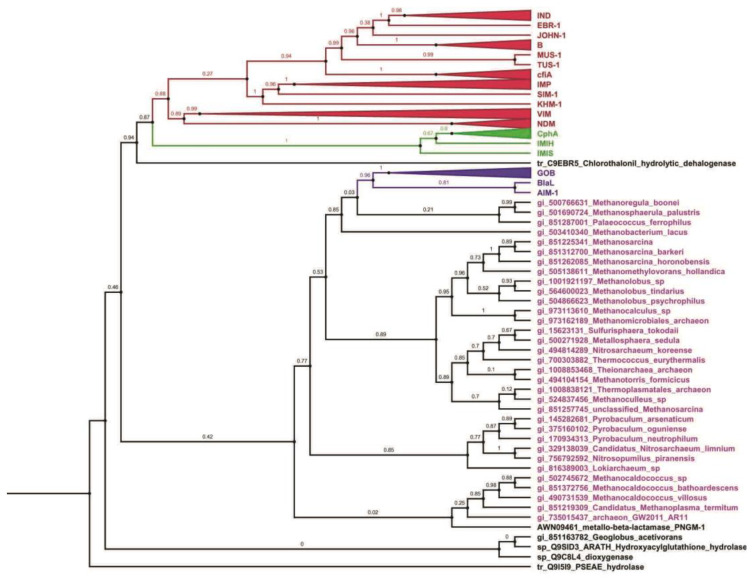
New phylogeny:this phylogenetic tree contains a total of 205 sequences (clades have been collapsed). The tree was constructed in FastTree and visualized in (midpoint rooted increasing order) FigTree. The color scheme of the leaves indicates that sequences belong to different groups/ families- Red, green, and blue indicate Metallo-β-lactamase B1, B2, and B3, respectively, while magenta color indicates archaeal sequences while black indicates diverse function. The complete figure can be seen in Figure S1.

Because the metallo-β-lactamases have the same fold, they likely arise from a common ancestor. Even if the metallo-β-lactamases fold is conserved, the identity level could be very low between the different members of this superfamily (less than 5%). The members share structural features such as the metallo-β-lactamase (MBL) fold and a mononuclear or binuclear active site center with a unique metal-binding motif (H-X-HX-D-H). Even in the functional group of β-lactamase, these sequences are highly divergent, for example only 11% of the amino acids are conserved between MBL B1 and B2, and only 9% are conserved between MBL B3 versus B1or B2. In addition, when aligning all the sequences, one or three amino acids will be shared (hopefully in the active sites). Because very few amino acids are conserved, the rate of phylogenetic artifacts increases due to the sharing of the same amino acid in the alignment. Thus, it is likely that the phylogenetic analysis will be robust at the subfamily level but not reliable between sub-families. We can also hypothesize that the sequences share a common ancestor, but the ancestor is so old that very few amino acids are found in common. In that case, we could define sub-groups based on network similarity, as described by Baier and Tokuriki [13] (of course, in that case, we have a phenetic analysis, and therefore the evolutionary history is only a guess). Because two different members belonging to the different superfamilies are not well conserved and because more than 30,000 sequences are, for example, available in the Pfam database, a phylogenetic tree is not possible, and a pre-classification must be conducted. This pre-classification can be obtained via the similarity network [14] that is the most comprehensive clustering on the β-lactamases fold described so far (Figure 1). This analysis, as well as other analyses [15], including ours [16,17,18], helped us to define several subfamilies of the metallo-β-lactamases, allowing us to perform precise analysis on some of them. We redefine the subfamily metallo-β-lactamase B3, glyoxalase 2, sulfur dioxygenase, metallo-β-lactamase B1/B2, TNP dehalogenase, and the archaea metallo-β-lactamase-like [13,15,16,17]. To perform a robust phylogenetic analysis, we used a distance-based phylogenetic analysis since very few positions are conserved (Figure 2).

The phylogenetic analysis shows that MBLB1, MBLB2, and TNP dehalogenase form a group while MBLB3 forms a group with the MBL-like archaea; the NJ analyses were unable to classify glyoxalase 2 sulfur dioxygenase families.

The other families include, for example, SNM, ribonuclease Z, B -CASP RNases [13,19].

### 2.3. Evolution of the Metallo-β-Lactamase Activity via an Increase in the Probability of Shift and Fixation of the New Character

The metallo-β-lactamase evolved via iso-convergent evolution due to the increase in the probability of shift (mutation rate) and due to the promiscuous activity. Most and maybe all enzymes, besides their physiological reaction, are capable of catalyzing secondary reactions termed promiscuous reactions [20,21]. The ratio *k*cat/*KM*, an index that is usually used for comparing the relative rates of an enzyme acting on the alternative substrate, shows a value around 10.5 to 10.8 M-1s-1 for the “native” substrate of a given enzyme, and lower activity of several orders for the promiscuous activities [8,13]. It was proposed by Jensen [22], more than forty years ago, that this ability to catalyze multiple chemically distinct reactions in addition to their primary function constitutes a functional repertoire from which the enzyme can be co-opted and further enhanced by mutations. The phylogenetic analysis above shows that the archaeal MBLforms a monophyletic clade with B3 metallo-β-lactamases. The archaea metallo-β-lactamase-like have a promiscuous activity [23]. The *k*cat/*K*M is around 20 in the case of nitrocefin while this activity is 10,000-fold higher in the case of MBL B3 β-lactamases l (B3 β-lactamase) [24,25,26,27]. The phylogenetic analysis also shows that TPN dehalogenases, which form a mono-phylogenetic group with MBL1 and B2, also display a promiscuous activity [13].

All the other families are less phylogenetically related, but some of them display lactamase activity, such as 1VJN (Uniprot ID: Q9WY50) family [13] whose physiological activity is unknown, and displays a good β-lactamase activity with a *k*cat/KM around 10,000. Furthermore, promiscuous activity is found in different structural families.

In the case of the glyoxalase 2 sulfur dioxygenase, TPN dehalogenases families PqqBalso display a promiscuous β-lactamase activity [13]. This is also the case for ribonuclease Z [13,18,28], MBLAC1 [17], as well as other enzymes with unknown function [13]. Therefore, promiscuous β-lactamase activity is found in most of the members of the metallo-β-lactamase superfamily and was likely to be present in the common ancestor of the superfamily. Thus, a bona fide β-lactamase activity evolved at least three times from promiscuous activity: in B1/B2 MBL, B3 MBL, and 1VJN.

### 2.4. The Metallo-β-Lactamases Evolved via Iso-Convergent Evolution Because Possibly Also of Positive Selection

Besides the increase in probability shift, the iso-convergence evolution of the β-lactamase activity is likely to be due to an increase infixation; in that case, positive selection can be a possibility. The secretion of antimicrobial compounds by microbes including lactamin could be an ancient strategy to improve the survival of microbes competing for space and nutrients with other microorganisms. Thus, the emergence of resistance mechanisms to antimicrobials could also be an ancient natural response process [29,30]. β-lactams are antimicrobials able to block the DD-peptidases involved in bacterial cell wall synthesis. Therefore, a mutation increasing the β-lactamase activity from a weak activity can be selected and the species having this activity will have an advantage over the others.

The β-lactams include five naturally occurring families. Four of them block the DD-peptidase: carbapenems, penicillin/cephalosporin, monocyclic β-lactams, and sulfazecin/monobactam. These families are synthesized by different biosynthetic pathways [31] and therefore evolved via allo-convergent evolution. It is possible that the β-lactamase activity evolved many times (from promiscuous activity) in response to one of these β-lactams synthesis pathways.

In the case of a mutation giving rise to an increase in the β-lactamase activity, two scenarios are possible: (i) a decrease in the original function, in which case the mutation will be counter selected; (ii) the original function is preserved, in which case the mutation will not be counter selected. In the first case, a gene duplication event could help with the fixation [32].

### 2.5. Iso-Convergent Evolution of the Serine β-Lactamase Family

As mentioned above, β-lactams inhibit penicillin-binding proteins(PBP), which are involved in the cell wall synthesis of bacteria by performing cross-linking of peptide chains to form peptidoglycan. The inhibition is done by acylating an active-site serine which is an essential penicillin-binding protein activity. PBPs were likely the precursors of the serine β-lactamases with the rate of deacylation being increased dramatically for serine β-lactamase family, compared to PBPs that exhibit a fast acylation step compared to a slow deacylation step. Formation of the PBP-acyl enzyme complex has half-lives ranging from around 10 min to more than 24 h depending on the PBPs and the β-lactam [33,34,35].

Some PBPs have evolved to function as weak β-lactamase with a slow turnover of the β-lactam substrate [36].For example, cefotaxime deacylation rates are 70to 80-fold higher for PBP2x variants than that for the wild-type enzyme in *Streptococcus pneumonia* [37]. Diene et al. showed [23] that archaea DD peptidase-like has a promiscuous β-lactamase activity (*k*cat/KM = 16.57 s^−1^ M^−1^). Therefore, the PBPs can develop the β-lactamases’activity in an iso-convergent manner, due in part to their β-lactamases’ promiscuous activity. The discussion concerning the positive selection and the role of duplication on the fixation of the events is the same as above.

### 2.6. Hypothesis: Allo-Convergent Evolution Should Be Linked to Iso-Convergent Evolution

Because of the promiscuous activity of the different protein folds (MBL lactamase fold and DD peptidase fold) positive selection and lack of constraint, both folds evolved a bona fide activity via allo-convergent evolution. Furthermore, inside each family the β-lactamase activity occurred not only one time but several times (iso-convergent evolution). We propose here that when allo-convergent evolution is evidenced, iso-convergent evolution should be also found. Of course, this will depend on the size of the superfamilies where the allo-convergent event occurs. Unfortunately, very few enzymatic families have been studied as well as the β-lactamase families. Another problem that prevented such observation is that authors do not make a clear distinction between allo-convergent and iso-convergent evolution even if the difference is sometimes mentioned by using the term of parallel evolution. However, as discussed by Pontarotti and Hue [1] the term is confusing, as some authors use this term to explain a morphological shift with the same genetic mechanisms.

Many cases of allo-convergent evolution have been reported [7]. One interesting case to start with could be the one of paraoxonase that evolves twice via allo-convergent evolution from two different folds, exhibiting ancestral promiscuous paraoxonase activity [38]. We propose that the paraoxonase activity also evolves inside each family and this can be tested.

Finally, the β-lactamases’ functional/structural evolutionary analysis allows the identification of two distinct evolutionary processes: exaptation and coevolution. In the case of MBL fold, we witness an exaptation. This term is used for a character that evolved a given function and is then used for another one [39], which is the case for the MBL lactamase. However, in the case of A, C, D serine β-lactamase we evidenced a specific case of exaptation that corresponds to a coevolutionary arms race (Van Valen, 1973). Indeed, the β-lactams used the DD peptidase active site to block the DD peptidase. The DD peptidase, in turn, evolved its hydrolysis activity against the β-lactams.

Furthermore, concerning the MBL fold based on our analysis, it is likely that the ancestral MBL fold had a hydrolase activity against an unknown organic compound and a promiscuous β-lactamase activity or other promiscuous hydrolase activity. This information is important regarding the understanding of the early phase of life evolution. Two main hypotheses aim to explain the earliest phases of evolution: the autotrophic and the heterotrophic origins. Theories for autotrophic origins propose that the first cells satisfied their carbon needs from CO_2_ [40], while heterotrophic origin theories propose that the first cells lived off the fermentation of reduced organic compounds present in some kind of rich organic soup [41]. β-lactamase fold function could have been important for the two scenarios. In the case of the heterotrophic hypothesis, the ancestral β-lactamase fold could have been involved in a hydrolysis reaction inorganic compounds already present on earth and was able to provide energy to the proto cell. In the case of autotrophic hypothesis, the next step could have been fermentations [42], and there the β-lactamase could have been extremely useful in such a process for its hydrolysis capacity of specific organic components. The promiscuous activity of ancestral MBL fold could have then evolved to a more efficient activity against new organic compounds found in the environment. Regarding the DD-transpeptidases (PBP)/serine β-lactamase families, they belong to a single clan (fold) with serine hydrolase properties [43], the enzymatic reaction of the PBP, which has the function ancestral to all of the group, already has a complex function D-alanine carboxypeptidase, peptidoglycan transpeptidase, and peptidoglycan endopeptidase. We do not have a hint about the previous functions, this prevents a discussion similar to that of the metallo-β-lactamase.

## 3. Materials and Methods

### 3.1. Sequence Selection

The protein sequences were collected from the ARG-ANNOT [44] and the NCBI database. A total of 205 protein sequences were considered in this study. These sequences belong to serine β-lactamases, metallo-β-lactamases, dehalogenase, dioxygenases, and archaeal encoded β-lactamases.

### 3.2. Functional Domain Identifications

The functional domains (proteins are generally composed of one or more functional regions, commonly termed domains) of these sequences were identified and extracted through NCBI Conserve Domain Database (CDD) [45] and Pfam release 32.0 database [46]. CDD offers both an archive of pre-computed domain annotations as well as live search services for both single protein or nucleotide queries and larger sets of protein query sequences. The Pfam database is a collection of protein families, each represented by multiple sequence alignments and hidden Markov models (HMMs). The Pfam and CDD were executed with the default settings on their respective websites/servers.

### 3.3. Phylogenetic Tree Construction

To explore the phylogenetic tree of the selected sequences, we performed multiple sequence alignments using the MUSCLE algorithms. Multiple sequence alignment trimming was performed using the trimAL [47], which removes poorly aligned regions. Next, the phylogenetic tree was constructed in FastTree [48] and visualized with FigTree (http://tree.bio.ed.ac.uk/software/figtree).

## Supplementary Materials

Supplementary materials can be found at https://www.mdpi.com/1422-0067/21/17/6260/s1.
