# Supplementary material for: Promiscuous Enzyme Activity as a Driver of Allo and Iso Convergent Evolution, Lessons from the β-Lactamases
Source: Int J Mol Sci. 2020 Aug 29;21(17):6260. doi: 10.3390/ijms21176260 (PMC7504333; doi:10.3390/ijms21176260)
Supplement: Supplementary file 1 [file ijms-21-06260-s001.pdf]

Supplementary data

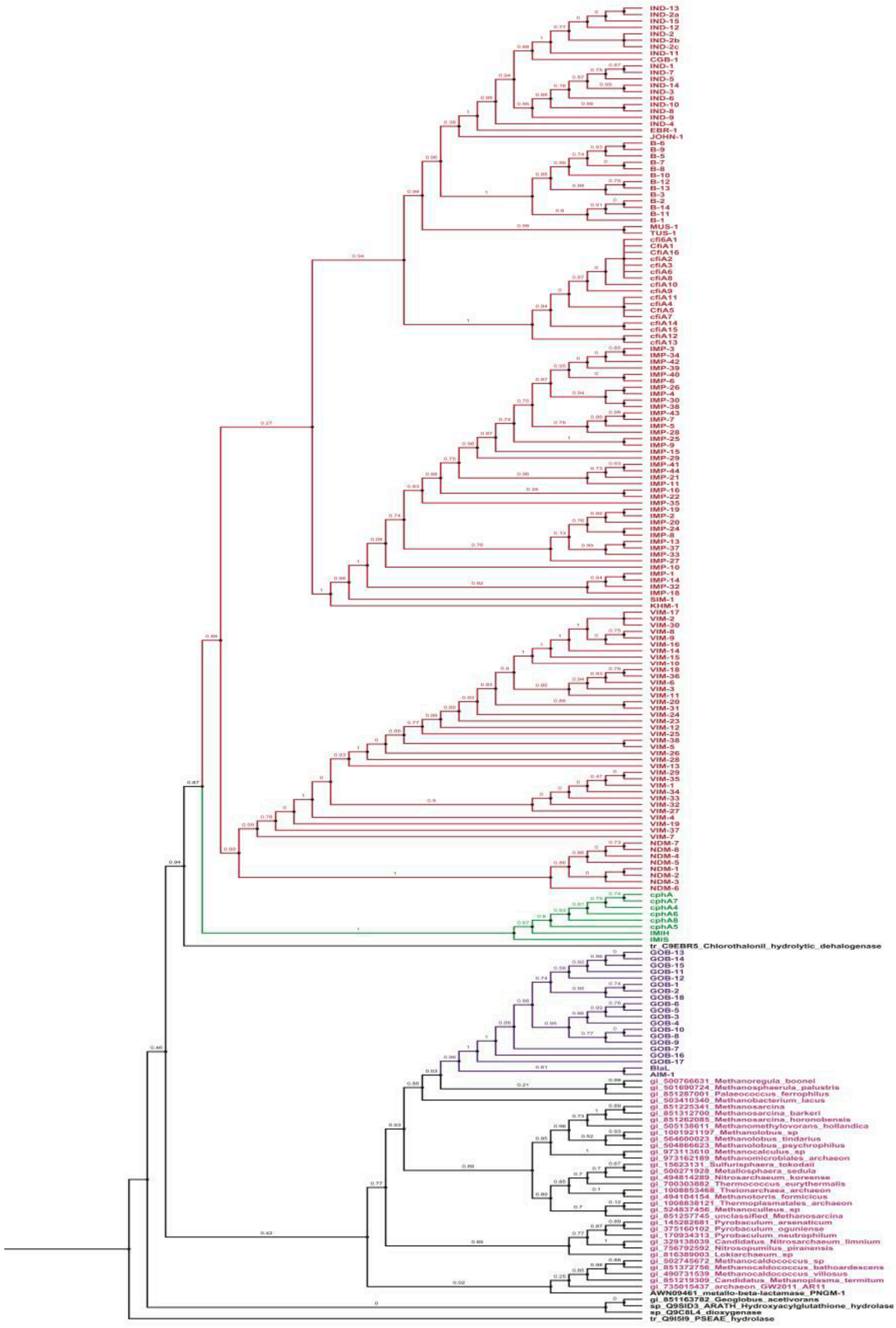

Figure S1: This phylogenetic tree contains a total of 205 sequences. The tree was constructed in FastTree and visualized in (midpoint rooted increasing order) FigTree. The coloring scheme of the leaves indicates sequences belongs to different group/ family- Red, Green and Blue indicates Metallo-β-lactamase B1, B2 and B3, Magenta color indicates archaeal sequences while black indicates diverse function.
